# CCR5 gene editing and HIV immunotherapy: current understandings, challenges, and future directions

**DOI:** 10.3389/fimmu.2025.1590690

**Published:** 2025-06-18

**Authors:** Jia-Wen Wang, Jia-Hui Liu, Jian-Jun Xun

**Affiliations:** Department of Orthopedics, The Fourth Hospital of Hebei Medical University, Shijiazhuang, Hebei, China

**Keywords:** HIV, CCR5, gene editing, immunotherapy, synergistic strategy, viral reservoir, challenges, future directions

## Abstract

Human immunodeficiency virus (HIV) infection remains a major global public health challenge. Although highly active antiretroviral therapy (HAART or ART) can effectively control viral replication, it fails to eradicate latent viral reservoirs and poses limitations such as lifelong medication and cumulative drug toxicity. This study focuses on the pivotal role of C-C chemokine receptor 5 (CCR5) gene editing in HIV immunotherapy, particularly highlighting the natural resistance to R5-tropic HIV strains observed in the “Berlin” and “London” patients carrying the homozygous CCR5-Δ32 mutation. We further explore the synergistic potential of multiplex gene editing strategies—including CCR5, CXCR4, and HIV LTR loci—and the combinatorial mechanisms between gene editing technologies and immunotherapy. A personalized treatment framework is proposed to address the clinical heterogeneity among people living with HIV. In addition, we assess the balance between long-term safety and global accessibility of gene-editing approaches such as CRISPR/Cas9, emphasizing strategies to enhance therapeutic efficacy while reducing cost and off-target effects. Our findings suggest that the integration of CCR5-targeted gene editing with immune-based interventions holds great promise for overcoming current therapeutic limitations and achieving functional HIV cure. However, key challenges—such as immune rejection, viral tropism switching, and economic feasibility—must be resolved. This integrative approach provides a robust theoretical and technical foundation for the next generation of HIV treatment paradigms.

## 1 Introduction

Human immunodeficiency virus (HIV) has exerted a profound impact on global public health, claiming millions of lives (1). Highly active antiretroviral therapy (HAART or ART) has significantly altered the natural course of HIV infection, prolonging survival and improving quality of life for those affected (1, 2). However, ART is not curative: it cannot eliminate latent viral reservoirs (3–5), necessitates lifelong adherence, and is associated with cumulative drug toxicity and the emergence of resistant viral strains (2, 6).

Traditional immune-based strategies—such as the use of broadly neutralizing antibodies (bNAbs) to target circulating virus (7–9) or immunostimulatory agents to enhance host immune responses (10, 11)—have shown promise but remain limited in their ability to eliminate latent HIV. Similarly, allogeneic hematopoietic stem cell transplantation (allo-HSCT) is constrained by high procedural risk and donor scarcity (6, 12).

The discovery of C-C chemokine receptor 5 (CCR5) as a major HIV co-receptor represents a breakthrough in overcoming these limitations. HIV entry into CD4^+^ T cells and other host cells requires not only CD4 receptor binding but also co-receptors such as CCR5 or CXCR4 (3, 13). Case studies of the “Berlin” and “London” patients—who achieved viral remission following transplantation from CCR5-Δ32 homozygous donors—have provided compelling evidence that genetic disruption of CCR5 can confer natural resistance to R5-tropic HIV strains (6, 12). These findings have catalyzed the rapid development of gene editing technologies targeting CCR5, including CRISPR/Cas9, for potential curative therapy (9, 13–15).

This paper seeks to address four key questions:

1. Synergistic Multi-target Editing: How can simultaneous editing of CCR5, CXCR4, and HIV LTR collectively establish a comprehensive viral blockade to counteract tropism switching and latent reactivation?
2. Gene-Immune Synergy: How can gene editing augment the anti-HIV capacity and persistence of immune cells? Conversely, how can immunotherapy complement gene editing to eradicate latent reservoirs more effectively?
3. Personalized Approaches for Clinical Heterogeneity: Given the high variability in viral subtypes, host immunity, and genetic background among HIV-infected individuals, how can we design broadly applicable yet individually adaptable treatment regimens?
4. Balancing Safety and Accessibility: How can we ensure long-term safety while enhancing global accessibility through technological optimization and innovative payment models?

**Figure 1:**
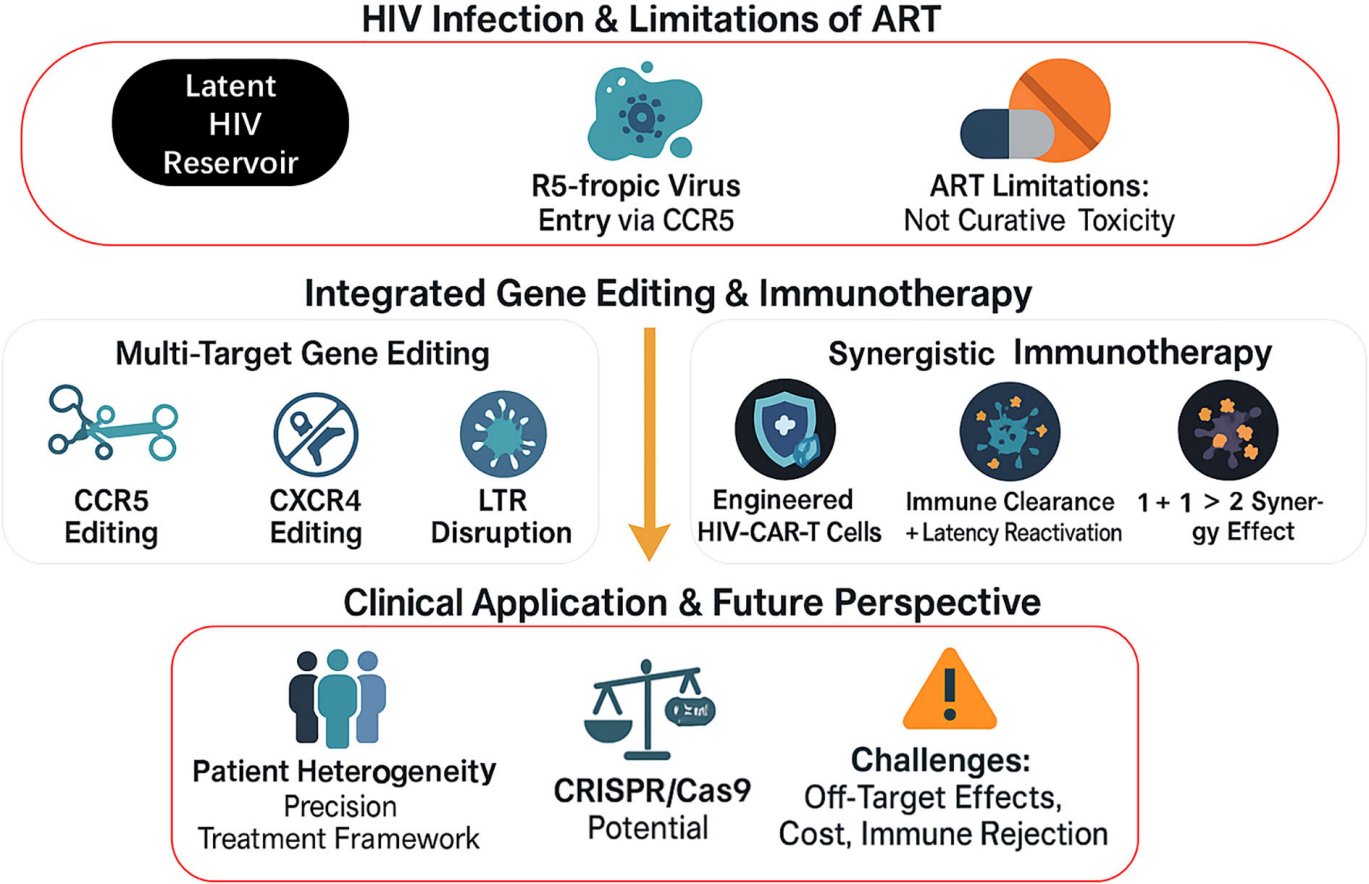
Integrated Strategy of Gene Editing and Immunotherapy for HIV Treatment. This figure presents an integrated framework for HIV treatment, structured in three levels from top to bottom: First, it outlines the characteristics of HIV infection and limitations of current antiretroviral therapy (ART); the middle section showcases two core therapeutic approaches—multi-target gene editing (CCR5, CXCR4, and LTR) and synergistic immunotherapy (HIV-CAR-T cells, etc.); the bottom displays clinical application considerations, including patient heterogeneity, CRISPR/Cas9 technology potential, and implementation challenges.

**Figure 1** outlines the integrated framework for HIV treatment, which systematically combines multi-target gene editing with synergistic immunotherapy, not only intervening at key stages of HIV infection but also addressing implementation challenges in clinical applications.

## 2 Current advances in integrating gene editing with immunotherapy for HIV

For R5-tropic HIV-1 strains—which dominate during the early and chronic phases of infection—C-C chemokine receptor 5 (CCR5) is an essential co-receptor for viral entry into CD4^+^ T cells and macrophages (3, 13). Its expression directly determines the susceptibility of these target cells to HIV. Individuals with naturally occurring CCR5 deletions, such as the homozygous CCR5-Δ32 mutation, exhibit high resistance to HIV-1 infection, providing a theoretical rationale for CCR5-targeted gene editing as a therapeutic strategy (6, 12).

In recent years, molecular tools including zinc finger nucleases (ZFNs), transcription activator-like effector nucleases (TALENs), and the clustered regularly interspaced short palindromic repeats (CRISPR)/Cas system have enabled precise targeting and editing of the CCR5 gene (14, 16, 17). Each technology offers unique features and therapeutic potential in the context of HIV treatment (see **Table 1**). Notably, CCR5 editing using CRISPR/Cas9 has progressed to early-phase clinical trials, including NCT03164135, which assessed CRISPR/Cas9-mediated CCR5 editing in hematopoietic stem cells for patients with both HIV and acute lymphoblastic leukemia—demonstrating feasibility and safety (18).

**Table 1:** Comparative characteristics of major gene editing technologies for CCR5-targeted HIV therapy.

Technology	Mechanism of Action	Advantages	Limitations and Challenges	Representative Studies/Advances	References
ZFNs	Custom-designed zinc finger proteins recognize specific DNA sequences and dimerize FokI nucleases to induce DNA cleavage.	One of the earliest technologies applied in CCR5 gene editing to enter clinical trials; has accumulated clinical data on safety and efficacy.	Design and construction are relatively complex; higher risk of off-target effects and potential immunogenicity.	The SB-728-T clinical trial demonstrated that autologous T cells edited by ZFNs and reinfused into patients yielded acceptable safety profiles and virological/immunological benefits (13, 30).	(3, 9, 13, 30)
TALENs	Transcription activator-like effector (TALE) proteins recognize specific DNA sequences, fused to FokI nucleases to cleave DNA.	Modular DNA-binding domains provide improved specificity over ZFNs, with relatively reduced off-target activity.	Construction remains technically demanding; large molecular size may hinder packaging and delivery via certain viral vectors.	Efficient CCR5 gene editing has been demonstrated (3); Schwarze et al. (2021) developed an automated, clinical-scale production system for TALEN-edited CD4+ T cells (15).	(3, 15)
CRISPR/Cas9	A single guide RNA (sgRNA) directs the Cas9 nuclease to specific genomic loci for site-specific double-strand breaks.	Easy to design and implement; high editing efficiency; allows for multiplex editing of several genes simultaneously.	Main safety concern is off-target effects, although various strategies exist to enhance specificity; PAM sequence dependency; long-term Cas9 expression may elicit immune responses.	Numerous *in vitro* and humanized mouse model studies confirm its high efficiency in CCR5 editing (14, 18, 37); early-phase clinical trials have begun exploring its application in HIV treatment (18).	(14, 17, 18, 25)
Base Editors (BE)	Fusion of Cas proteins (typically nCas9 or dCas9) with nucleotide deaminases enables precise single-nucleotide substitutions without introducing double-strand breaks.	Enables precise base conversions while avoiding risks associated with DSBs, such as indels and chromosomal translocations.	Potential off-target base editing (both DNA and RNA); limited editing window constrains targetable base positions.	Jia et al. (2025) successfully edited the PD-1 gene using CE-8e-SpRY mRNA base editors delivered via LVLPs under a “Gag-only” packaging strategy (25).	(25)

Chronic viral infections such as HIV, HBV, and HCV share the common hallmark of progressive T cell exhaustion, which is closely linked to sustained expression of immune checkpoint molecules like programmed cell death protein 1 (PD-1), PD-L1, and cytotoxic T-lymphocyte-associated protein 4 (CTLA-4) (19). Evidence suggests that PD-1/PD-L1 blockade may restore the function of HIV-specific CD8^+^ T cells, improving their ability to clear infected cells and potentially reactivating latent reservoirs (20, 21). Additionally, anti-PD-1 chimeric antigen receptor (CAR) T cells have shown efficacy in targeting SIV-infected CD4^+^ T cells in germinal centers of non-human primate models (19), raising interest in the use of immune checkpoint inhibitors in HIV treatment.

Although CCR5 gene editing and immunotherapies—such as checkpoint inhibitors and CAR-T cells—have each demonstrated distinct promise, studies combining these approaches in a synergistic and systematic manner remain limited. Current research often involves engineering CAR-T cells (21–24)to co-express anti-HIV shRNAs or edited CCR5 genes (21, 22, 24), primarily to enhance the therapeutic durability of the immune cells. Similarly, allogeneic HIV-specific CAR-T cells engineered to secrete PD-1-blocking scFv have shown increased cytotoxicity against HIV Env^+^ cells (13), but such efforts still focus on incorporating immune-modulatory elements into CAR-T cell functionality rather than exploring dynamic synergy between gene editing and immunotherapy.

Most existing studies remain centered on either optimizing gene editing efficiency and safety (7, 15, 25) or developing novel immune-based therapies for HIV (4, 10, 11, 19, 22, 24, 26–29), with few addressing their integrated or synergistic potential.

## 3 Cutting-Edge Developments

Due to the high genetic variability of HIV, single-target CCR5 editing strategies alone are insufficient. Thus, recent innovations in the field are moving toward two main directions: (1) constructing a comprehensive viral defense through multi-target gene editing, and (2) enhancing viral clearance through synergistic integration of gene editing and immunotherapy.

### 3.1 Multi-target gene editing to construct a comprehensive viral barrier

Following effective CCR5 disruption, HIV may switch coreceptor usage to CXCR4 (X4-tropic strains), enabling continued infection (30). Furthermore, once HIV integrates into the host genome, the virus can be reactivated via the long terminal repeat (LTR) region, which contains strong promoter and enhancer elements. This allows viral reactivation even in cells lacking CCR5 or CXCR4 expression (31, 32). Activation of LTR promotes Gag expression, enhances viral particle assembly (33), and facilitates reverse transcription (34), followed by integrase-mediated insertion of viral DNA into host chromosomes (35), ultimately driving viral replication (36).

Thus, multi-target gene editing strategies—targeting both host and viral genes—are critical for combating tropism switching, latent reactivation, and escape mutations. ZFNs and TALENs can be paired for multi-locus editing, as demonstrated by Schwarze et al. (2021), who achieved efficient CCR5 editing using TALENs (15). The CRISPR/Cas9 system offers more versatility by co-delivering Cas9 with multiple single-guide RNAs (sgRNAs) targeting CCR5, CXCR4, HIV LTR, and viral structural genes (e.g., *Gag*, *Pol*) (14, 18).

Simultaneous knockout of CCR5 and CXCR4 prevents infection by both R5- and X4-tropic viruses. Editing the HIV LTR suppresses transcriptional activation, while targeting *Gag* disrupts particle assembly. Collectively, such multi-site CRISPR/Cas9 interventions show superior efficacy in inhibiting viral replication and transmission (17).

The CRISPR/Cas12 system—specifically Cas12a (formerly Cpf1)—has unique features such as recognition of TTTN PAM sites and sticky-end cleavage. A crRNA array can generate multiple mature crRNAs for multiplex editing. This makes Cas12a well-suited for simultaneous targeting of diverse loci. Additionally, base editors (BEs) and prime editors (PEs) offer precise nucleotide modifications or small insertions/deletions without inducing double-strand breaks, minimizing the risk of chromosomal translocations and large deletions.

Jia et al. (2025) used lentiviral-like particles (LVLPs) to deliver CE-8e-SpRY mRNA, an adenine base editor, effectively targeting the PD-1 gene—highlighting both the precision and delivery feasibility of such systems (25). Given the differences in editing efficiency and off-target risks among platforms, combinatorial multi-platform strategies are encouraged, as they can cover over 90% of HIV strains and significantly enhance therapeutic efficacy (see **Table 2**).

**Table 2:** Quantitative on-target efficiency and off-target profiles of gene editing platforms.

Platform	Target Cells/Model	On-target Efficiency (% mean ± SD)	Off-target Detection	Representative References
ZFN (SB-728-T)	Peripheral CD4^+^ T cells (Phase 1 trial)	20–44%	≥1% off-target sites detected	(60, 61)
CRISPR/Cas9	Primary CD4^+^ T cells	27.3 ± 6.7%	<0.5%	(62)
CRISPR/Cas9 + RNP	Mobilized CD34^+^ HSPCs	81 ± 4%	Undetectable	(63)
CRISPR-HSPC Transplantation	Patient-derived peripheral CD4^+^ T cells	≈5%	No SAE; WGS revealed no significant off-target effects	(43)
Cas12a / BE4	CD4^+^ T cells / HSPCs	35–60%	No sites >0.1% by GUIDE-seq	(64, 65)

### 3.2 Synergistic integration of gene editing and immunotherapy

The concept of gene-immune synergy leverages the long-term protection conferred by gene editing and the potent viral clearance enabled by immunotherapy, aiming for a “1 + 1 > 2” therapeutic outcome. This can be achieved through two key pathways:

First, immune effector cells such as T cells and NK cells can be genetically modified using CRISPR/Cas9 to enhance their resistance to HIV infection. For example, when engineering HIV-targeted CAR-T cells, concurrent CCR5 knockout can protect them from HIV-mediated depletion post-infusion, extending their *in vivo* persistence and enhancing antiviral durability (21, 24). Studies have shown that such modifications significantly reduce viral load and may directly contribute to reservoir clearance (see **Table 3**). Current research is increasingly focused on generating CAR-T cells with dual functions: intrinsic HIV resistance and active antiviral cytotoxicity (37).

**Table 3:** Preclinical evidence for gene-immune combination therapies.

Combination Strategy	Model and Design	Antiviral Suppression/Clearance Outcome	Reference
CRISPR CCR5 + HIV-1 LTR-Gag Dual Targeting	ART-withdrawn humanized BLT mice (n = 23)	9/23 showed undetectable levels in ddPCR and VOA across multiple tissues within 10 weeks	(13)
CCR5-KO CD4^+^ T cells + Long-acting bNAb	Humanized BLT mice post CCR5-KO HSPC reconstitution; AAV6-delivered VRC07-523LS	Plasma HIV-RNA ≤ LOD (Day 28); no integrated proviral DNA detected in tissues	(66)
CCR5-KO HSPCs + PD-1 Blockade (in vitro)	CD34^+^ HSPCs edited via CRISPR, differentiated to CD4^+^ T cells with anti–PD-1 (10 µg/mL)	HIV-p24 decreased by 68% ± 5 at 72h post-infection (MOI 0.1)	(13)

Second, the combination of gene editing and immune checkpoint modulation can further improve viral eradication. Allogeneic HIV-specific CAR-T cells engineered to secrete PD-1-blocking scFv exhibited heightened cytotoxicity against HIV Env-expressing cells (13), demonstrating a clear case of synergy. Additionally, stem cell-derived CAR-T cells have shown effective migration and infiltration into viral reservoirs located in germinal centers, the central nervous system, and gut-associated lymphoid tissue in macaque models (22), offering strong experimental support for this strategy.

## 4 Challenges and limitations

As a retrovirus, HIV presents formidable challenges to therapeutic strategies due to its high mutation rate and ability to establish latent reservoirs. Certain HIV strains—particularly X4-tropic variants—can utilize CXCR4 as a coreceptor to enter host cells. This tropism shift from R5 to X4 is especially common in late-stage disease or after CCR5-targeted interventions (3). Furthermore, regulatory elements within the HIV long terminal repeat (LTR) region can independently drive viral transcription and replication, posing a risk of reactivation even in the absence of coreceptor expression.

Latent HIV reservoirs are anatomically dispersed throughout various tissues and cell types, including lymph nodes, spleen, gut-associated lymphoid tissue (GALT), and the central nervous system (CNS) (1, 3–5). These reservoirs predominantly reside in resting memory CD4^+^ T cells but also include macrophages and dendritic cells, which exhibit low metabolic activity, long half-lives, and resistance to conventional antiretroviral therapy (ART) (4, 12). The complexity and heterogeneity of HIV persistence across tissue compartments represent a major barrier to viral eradication (4, 5).

Moreover, HIV-infected individuals exhibit significant heterogeneity in terms of viral load, subtype, genotype, resistance history, CD4^+^ T cell counts, immune activation, and host genetic background (27). Such variability complicates treatment response, as a uniform therapeutic approach may not achieve comparable efficacy across different patients. In cases of severe immunosuppression, gene-edited cells may lack adequate immune surveillance support, diminishing their antiviral potential. This underscores the urgent need for diverse, personalized therapeutic strategies (1).

Although gene editing technologies—such as CRISPR/Cas9—have made substantial progress in improving target specificity, off-target effects remain a critical safety concern (14, 16, 17). The long-term *in vivo* persistence, genetic stability, and potential late-onset adverse effects of edited cells must be rigorously evaluated through large-scale, longitudinal clinical trials. These safety issues highlight the necessity of long-term surveillance frameworks (38).

Additionally, the economic burden of gene editing is considerable. One-time gene therapies currently cost 5–10 times more than conventional treatments (see **Table 4**) (30). This high upfront cost and limited scalability constitute significant barriers to widespread implementation, especially in low-resource settings.

**Table 4:** Cost-safety comparison between ART and one-time gene-immune therapy.

Indicator	Standard ART (INSTI-based)	Gene-Immune One-Time Therapy (Predicted / Approved Analogues)
Annual Drug Cost (US AWP)	36,000–48,000 USD/person·year (67)	2.8 M USD (Zynteglo); 4.25 M USD (Lenmeldy) (51, 52)
10-Year Accumulated Cost	360,000–480,000 USD (67)	2.8–4.25 M USD (single payment; installment or outcome-based models possible) (68)
Grade 3/4 Adverse Event Rate	≈10% (69)	6% (61)
Major Toxicities	Metabolic disorders, hepatic/renal impairment, weight gain (69)	Transient cytopenia, low-grade fever; no detectable off-target effects (61)
Adherence Requirement	Lifelong daily oral therapy (67)	Single infusion/transplant; routine post-procedure follow-up (68)
Accessibility	Widely available; generic annual cost ≈75 USD (67)	Limited to high-income countries; affordability challenges remain (70)

## 5 Discussion

Over the next five years, research will likely focus on strengthening the scientific basis for gene–immune synergistic therapies and refining preliminary treatment paradigms. Current humanized mouse models (2, 26, 37, 39, 40) and non-human primate models (7, 8, 22, 41) provide critical platforms for evaluating novel strategies. Safer and more efficient delivery systems—such as Gag-only lentiviral-like particles (LVLPs) (25) and optimized adeno-associated viral vectors (AAVs) (18)—should be explored in early-phase clinical trials using stepwise dose escalation and real-time safety monitoring to validate CCR5-targeted gene–immune combinatorial strategies, adhering to a “safety-first” principle (42).

Lessons learned from the first-in-human CRISPR-HSPC transplantation trial, which reported no serious adverse events during 19-month follow-up (43), can inform early efficacy assessments and data collection for future trials. Moreover, genetic heterogeneity among HIV patients must be considered: CXCR4-tropic viruses are associated with poorer ART response and accelerated CD4^+^ decline (44), while protective alleles such as HLA-B57 (45) and homozygous CCR5Δ32 (46) correlate with better viral control. Thus, stratified trials that enroll patients based on viral tropism, baseline viral load, and immune status are essential.

Promising preclinical findings—such as duoCAR-T cells eliminating over 90% of HIV-infected cells in humanized mice (47), PD-1 blockade reducing viral reservoirs and enhancing CD8^+^ T cell function in macaques (48), and dual-CRISPR strategies (CCR5 + LTR-Gag) achieving complete viral clearance in 39% of BLT-mice (49)—suggest that deeper investigation of combinatorial approaches (e.g., CCR5 editing + multi-specific CAR-T or CCR5 editing + PD-1 blockade) is warranted.

Patient preferences regarding the risk–benefit tradeoffs of gene therapy can be quantitatively integrated into clinical pathway design (50), supporting the development of truly individualized treatment strategies (41). To facilitate broad translation, future protocols must be optimized not only for biological efficacy but also for clinical feasibility and acceptability.

In the next decade, the high cost of gene therapy will necessitate parallel efforts to build scalable, cost-efficient manufacturing platforms. Approved therapies such as β-thalassemia gene treatments currently range from $2.8 to $4.25 million per patient (51, 52), often exceeding the annual health expenditure of many low- and middle-income countries. Current payment models rely on large, one-time upfront payments (53), underscoring the need for cost reduction.

Strategies such as serum-free suspension cultures, high-density perfusion, continuous chromatography, and optimized transfection workflows can reduce lentiviral vector production costs by up to 50% (54–56). Payment innovations—such as outcomes-based agreements and installment plans—could further improve affordability (57).

Importantly, technical breakthroughs must be accompanied by robust ethical and regulatory oversight, lifelong follow-up systems (58), and multi-stakeholder coordination to ensure sustained monitoring (3). Continued innovation in low-cost vectors, miniaturized CRISPR platforms (59), and automated manufacturing—coupled with forward-thinking reimbursement models—will be essential for overcoming current barriers and enabling widespread implementation of gene–immune strategies in HIV treatment.

## Data Availability

The original contributions presented in the study are included in the article/supplementary material. Further inquiries can be directed to the corresponding author/s.
